# Increased PAFAH1B3 was associated with poor prognosis and T-cell exhaustion microenvironment in hepatocellular carcinoma

**DOI:** 10.1007/s12672-023-00845-6

**Published:** 2023-12-08

**Authors:** Genhao Zhang

**Affiliations:** Department of Blood Transfusion, The First Affiliated Hospital of Zhengzhou University, Zhengzhou, 450052 China

**Keywords:** T-cell exhaustion, TEX, HCC, PAFAH1B3, PD1/PD-L1

## Abstract

The link between T-cell exhaustion (TEX) and PAFAH1B3 in hepatocellular carcinoma (HCC) remains unknown, even though both of them are related to overall survival. PAFAH1B3 expression was investigated in TCGA and ICGC data, and 50 paired clinical tissue section samples were used for qRT-PCR and immunohistochemistry (IHC) confirmation. The Immunocell Abundance Identifier (ImmuCellAI) was used to precisely calculate the abundance of TEX, and its accuracy was verified by single-cell RNA-seq and labeling of CD8 + T cells in clinical tissue sections. The IMVigor 210 cohort was used to demonstrate the predictive value of PAFAH1B3 for immunotherapy efficacy. Increased PAFAH1B3 is a standalone effector of poor prognosis in HCC patients. Patients who had greater PAFAH1B3 levels had more TEX infiltration. PAFAH1B3 expression was increased in TEX in the single-cell RNA-seq data. Patients with high PAFAH1B3 expression were more likely to respond favorably to PD1/PD-L1 treatment. In conclusion, PAFAH1B3 is closely related to TEX in the tumor microenvironment (TME) and can be a useful indicator for PD1/PD-L1 therapy.

**Supplementary Information:**

The online version contains supplementary material available at 10.1007/s12672-023-00845-6.

## Introduction

A critical immune cell in the tumor microenvironment (TME), CD8 + T lymphocytes are crucial for the destruction of tumor cells. When activated by tumor antigens at a sustained high frequency, CD8 + T lymphocytes within a tumor become dysfunctional and are unable to successfully eradicate cancer cells. T-cell exhaustion (TEX) is the name given to this syndrome [1]. In hepatocellular carcinoma (HCC), TEX-enriched patients had a worse overall survival rate than TEX-depleted ones [2]. Additionally, TEX causes significant epigenetic remodeling that renders these cells phenotypically stable and inhibits immune checkpoint inhibitors' effectiveness by blocking T cell renewal through immune checkpoint blockade (ICB) [3, 4]. Notably, TEX showed elevated and persistent PD1 expression, which sparked the creation of ICB treatments that specifically target PD-1 and its receptor PD-L1 [5]. To increase the effectiveness of immunotherapy and enhance patient prognosis, it is thus extremely special to understand the fundamentals of basic T cell immunity, understand the molecular processes of TEX, and create TEX-related biomarkers for screening responders and non-responders to immunotherapy.

Platelet-activating factor acetylhydrolase 1B3 (PAFAH1B3), one of the catalytic subunits of PAFAH, is essential for the growth, metastasis, angiogenesis, and development of drug resistance in cancer [6]. Multiple myeloma patients' bone marrow aspirates had higher PAFAH1B3 expression, which is significantly linked to a poor prognosis [7]. PAFAH1B3 knockdown decreases the proliferation, migration, and activation of oncogenic signaling in gastric cancer [8], osteosarcoma [9], lung adenocarcinoma [10], and hypopharyngeal squamous cell carcinoma [11]. This phenomenon is also confirmed in HCC [12]. Unfortunately, no studies have yet reported whether there is some link between TEX and PAFAH1B3 in HCC, which warrants further exploration.

Through a combination of bioinformatics analysis and clinical trial validation, we discovered for the first time in the current study that TEX and PAFAH1B3 had a substantial association and that PAFAH1B3 may be a predictor of ICB therapy.

## Methods

### Samples acquisition

A total of 927 HCC samples were used in this study, including 342 from The Cancer Genome Atlas-LIHC cohort (TCGA, https://xenabrowser.net/), 260 from the LIRI-JP cohort (ICGC, https://dcc.icgc.org/), 10 from the GSE149614 cohort, 320 from the IMvigor210 cohort and 50 from the HCC clinical samples. In Supplemental Table S1, data on the pathology of HCC patients was shown. Samples with insufficient clinicopathological traits or 30-day survival rates were ruled ineligible. To make up for the dearth of normal sample instances in the TCGA dataset, 110 normal samples from the GTEx dataset were employed. The log2 (FPKM + 1) transformation was used to standardize the transcriptome data. The batch effects between the normalized data from the TCGA and GTEx were corrected using Combat from the R package "SVA." To accurately assess the quantity of TEX, the immune cell abundance identifier (ImmuCellAI, 2020.02) was used [13].

### qRT-PCR and immunohistochemistry (IHC) assay

As was done before [14], the qRT-PCR method was used to ascertain the mRNA expression of the genes covered in this investigation among tumor tissues and corresponding paracancerous tissues from 50 HCC patients. Primer sequences are displayed in Table S2. IHC tests using anti-PAFAH1B3 (Proteintech, China) or anti-CD8 (Abcam, UK) antibodies were separately performed on paraffin-embedded tissues from HCC patients. The slides were incubated with secondary antibodies and imaged using a Leica DM 2500 microscope. The immunostaining intensity of the required proteins was evaluated and graded separately by two distinct observers. To differentiate between low/loss (≤ 4) and high (> 4) expression of PAFAH1B3 in HCC and corresponding paracancerous tissues, a final score derived from the sum of the extent of expression score (no positive cells = 0, < 10% = 1, 10–50% = 2, positive staining of > 50% = 3) and intensity score (negative = 0, weak = 1, moderate = 2, strong = 3) was employed.

### Assay for double-label immunofluorescence

The TSA Fluorescence Kit was utilized to conduct double-labeled immunofluorescence staining on the aforementioned HCC tissue samples. In summary, tissue slices were pre-stained with CY3 and Alexa Fluor 488 after being treated with primary antibodies anti-PAFAH1B3 (Proteintech, China) and anti-PD1 (Abcam, UK). After that, CaseViewer software was used to scan and analyze the parts. Lastly, the degree of PAFAH1B3 + PD1 + cell infiltration in HCC tissue samples was noted.

### TEX identification in the single-cell sequencing data

Quality control and cell screening were carried out on the GSE149614 dataset using the Seurat package [15] as reported by the previous study [16], yielding a total of 31,396 cells and 17 distinct subgroups for additional analysis. These distinct groupings were identified based on several marker genes (Figure S1). We picked out each NK/T cell separately and binned them once again with dim = 50 and resolution = 0.1. Finally, TEX was identified according to the TEX marker (HAVCR2, TIGIT, CTLA4, LAG3, and PDCD1).

### The pathway richness analysis

To assess the variation in biological processes between the PAFAH1B3-high and PAFAH1B3-low subgroups in the TGCA cohort, we used GSVA analysis with the R package 'GSVA'.

### Comprehensive analysis of the effectiveness of immunotherapy

We looked at the differences in gene expression between the PAFAH1B3-high and PAFAH1B3-low subgroups for numerous immune checkpoint inhibitors (ICIs). The prognostic utility of PAFAH1B3 for immunotherapy was further demonstrated using an anti-PD1/PD-L1 inhibitor cohort (IMVigor 210) with relatively extensive transcriptome data and information on immunotherapy response.

### Potential tumor-sensitive drug prediction

We especially looked at the connections between 216 medicines in the CellMiner database and PAFAH1B3 expression [17]. A drug is considered tumor-sensitive if it has a Pearson correlation coefficient of more than three-tenths and its adjusted P-value is less than one in a thousand. The half-maximal inhibitor dosage (IC50) of the targeted medication was then predicted using gene expression levels to demonstrate therapeutic sensitivity. Furthermore, to confirm the effect of the screened tumor-sensitive drugs on PAFAH1B3, we constructed PAFAH1B3 low-expressing cell lines according to the previous study [12], which were incubated with tumor-sensitive drugs at 100% humidity, 37 °C, 5% CO2 in the recommended fetal bovine serum containing 10% fetal bovine serum (FBS, Sangon Biotech, China) in DMEM medium (Sangon Biotech, China) for colony formation assay. The colonies were fixed and stained with crystal violet (Sangon Biotech, China) in 10% ethanol for 5 min. Finally, the cell colonies were imaged and counted.

### Statistical analysis

R-4.2.1 performed all the statistical analyses. For quantitative data, the Wilcoxon rank sum test was used to analyze the statistical significance of variables with non-normal distributions, while the Student's t-test was used to check the statistical significance of variables with a regular distribution. All comparisons were two-sided with an alpha level of 0.05, and the false discovery rate (FDR) for multiple hypothesis testing was corrected using the Benjamini–Hochberg method.

## Results

### Increased PAFAH1B3 is an independent effector of poor prognosis in HCC patients

**Fig. 1:**
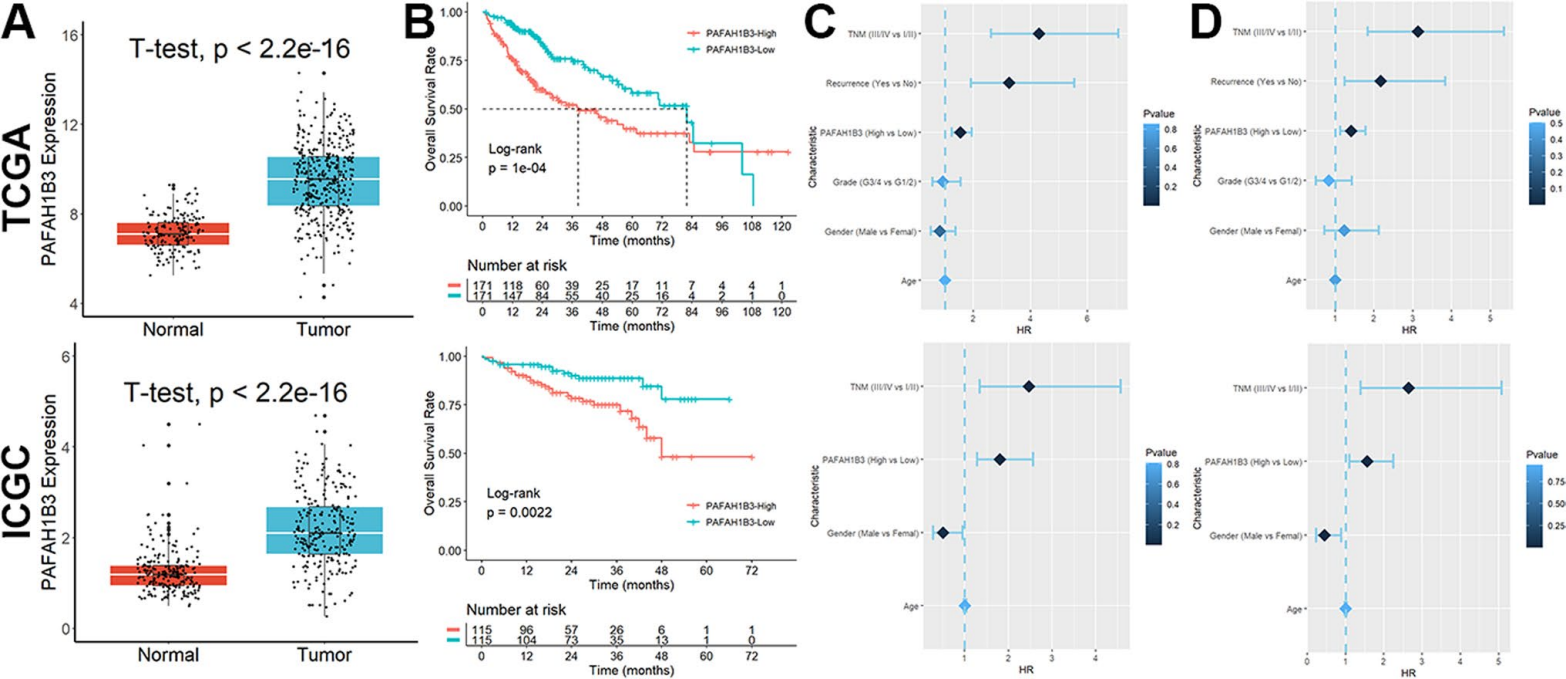
Increased PAFAH1B3 is an independent effector of poor prognosis in HCC patients. **A** Both in the TCGA and the ICGC cohort, PAFAH1B3 expression in tumors was noticeably greater than that in normal tissues. **B** Patients who expressed high levels of PAFAH1B3 had worse survival rates than those who expressed low levels of PAFAH1B3. **C** Univariate Cox regression analysis. **D** Multivariate Cox regression analysis

**Fig. 2:**
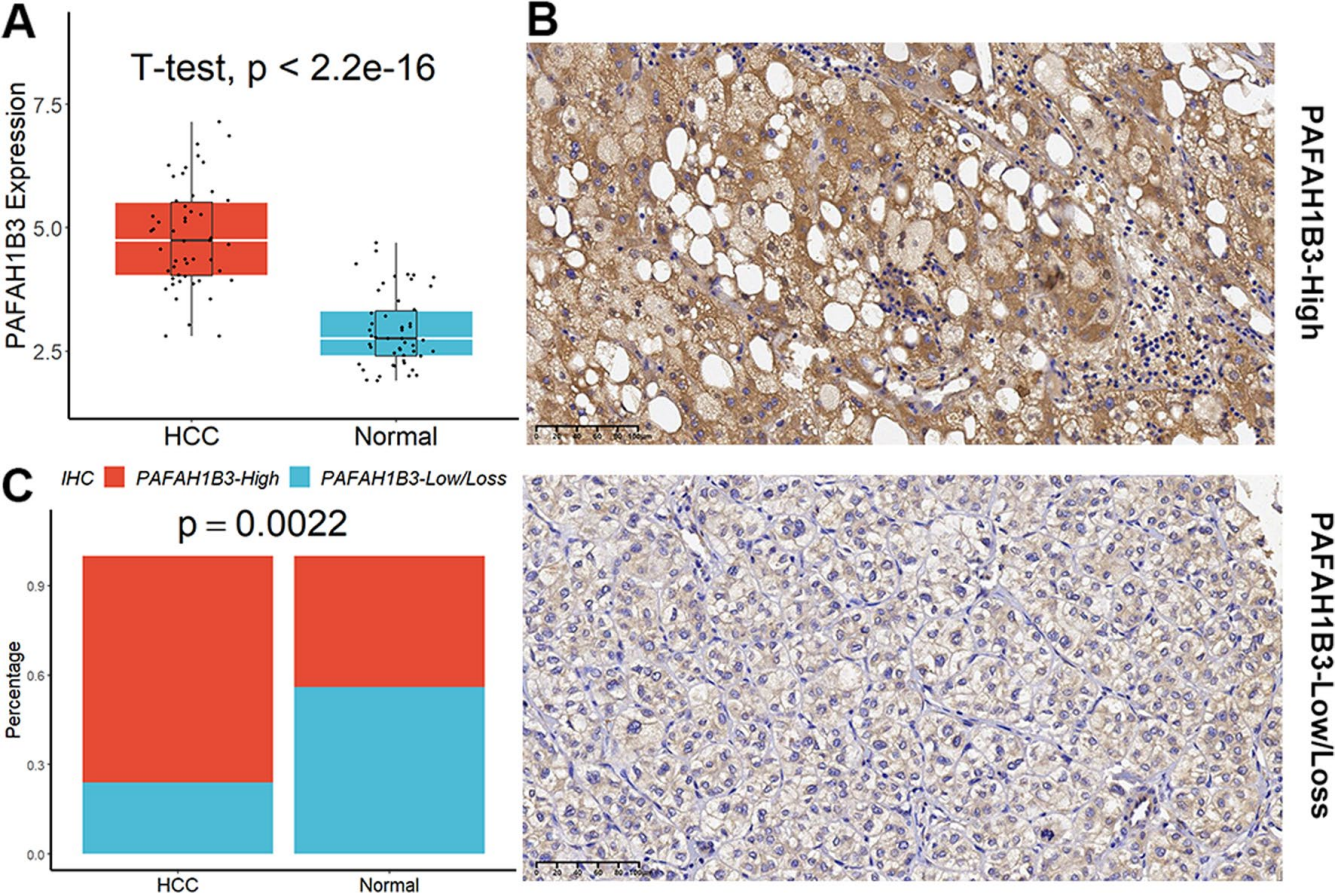
PAFAH1B3 expression in clinical samples. **A** In comparison to normal samples, HCC samples have considerably increased levels of PAFAH1B3 mRNA. **B** The expression of the PAFAH1B3 protein in HCC was variable. **C** The positivity rate of PAFAH1B3 protein was higher in HCC patients

Both in the TCGA and the ICGC cohort, PAFAH1B3 expression in tumors was noticeably greater than that in normal tissues (Fig. 1A). Patients who expressed high levels of PAFAH1B3 had worse survival rates than those who expressed low levels of PAFAH1B3 (Fig. 1B). According to univariate Cox regression analysis (Fig. 1C) and multivariate Cox regression analysis (Fig. 1D), increased PAFAH1B3 was a distinct effector in HCC patients who had a poor prognosis. Furthermore, high alpha-fetoprotein (AFP) levels, vascular invasion, later grade, later TNM stage, and recurrence were all substantially correlated with elevated PAFAH1B3 in HCC patients (Figure S2). The expression of PAFAH1B3 was then confirmed in clinical samples. In comparison to normal samples, HCC samples have considerably increased levels of PAFAH1B3 mRNA (Fig. 2A). The expression of the PAFAH1B3 protein in HCC was variable (Fig. 2B), and the positive rate was greater in tumor tissues than in normal tissues (Fig. 2C). Finally, the pathway richness analysis showed that samples with high PAFAH1B3 expression were mostly enriched in pathways related to cell cycle and mitosis, while samples with low PAFAH1B3 expression were primarily enriched in terms related to amino acids and energy metabolism (Figure S3).

### Patients with increased PAFAH1B3 had a higher proportion of TEX infiltration

**Fig. 3:**
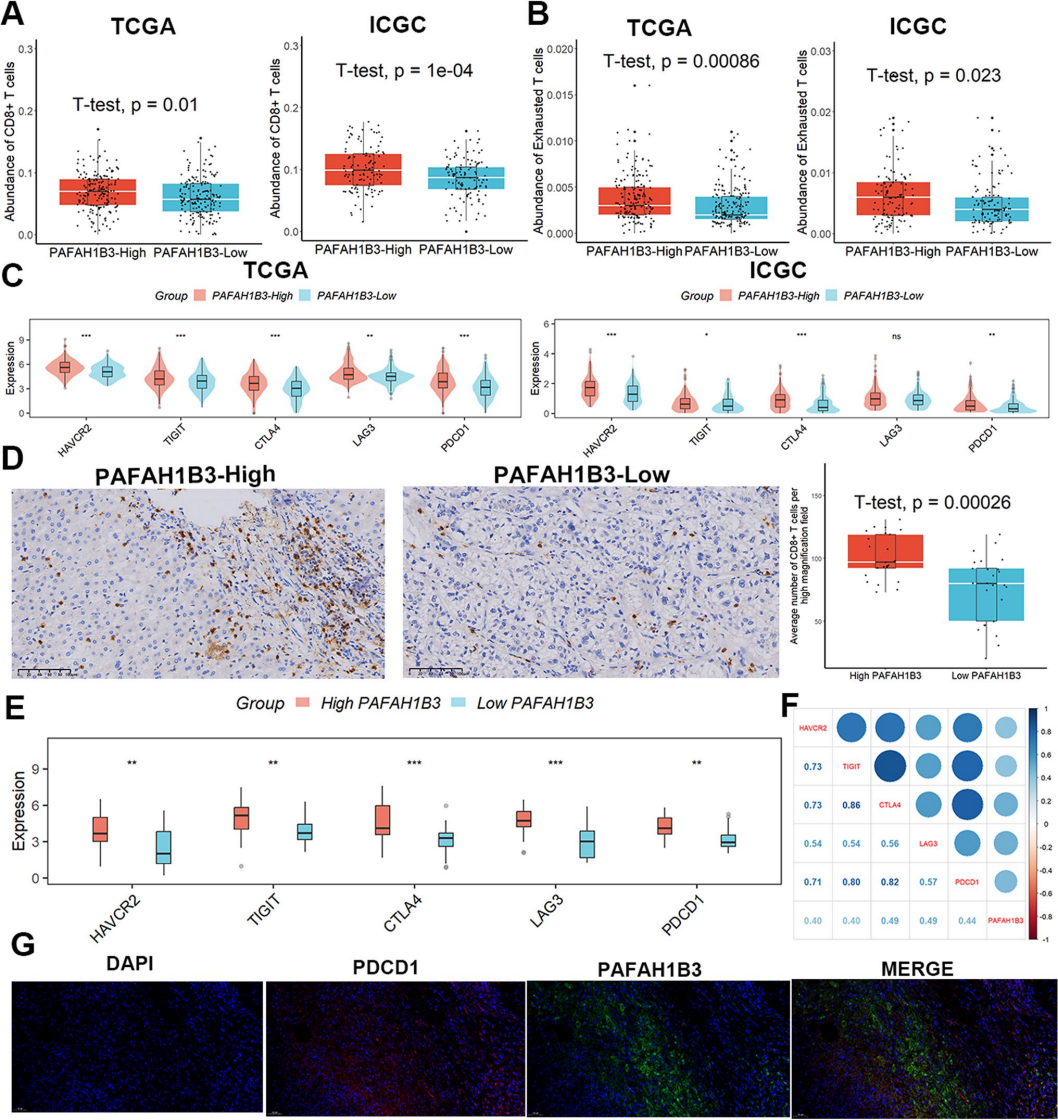
Patients with increased PAFAH1B3 had a higher proportion of TEX infiltration. **A**, **B** Samples with high PAFAH1B3 expression showed considerably more CD8 + T cell and TEX infiltration than samples with low PAFAH1B3 expression. **C** Although there was no discernible difference in LAG3 expression levels between the two groups in the ICGC cohort, the expression of these genes was higher in the samples with high PAFAH1B3 than in those with low PAFAH1B3. **D** Patients with high PAFAH1B3 expression had a larger percentage of CD8 + T cells infiltrated than those with low PAFAH1B3 expression clinical samples. **E** Expression of all TEX marker genes was considerably higher in these patients. **F** The expression of PAFAH1B3 was significantly and positively correlated with the expression of TEX-related marker genes. **G** Results of immunofluorescence staining. ns, not significant. *p < 0.05; **p < 0.01; ***p < 0.001

According to the findings of the ImmuCellAI analysis, samples with high PAFAH1B3 expression showed considerably more CD8 + T cell infiltration than samples with low PAFAH1B3 expression (Fig. 3A), in addition to having a larger abundance of TEX infiltration (Fig. 3B). The expression levels of the TEX marker genes HAVCR2, TIGIT, CTLA4, LAG3, and PDCD1 were also compared between the two groups. Although there was no discernible difference in LAG3 expression levels between the two groups in the ICGC cohort, the expression of these genes was higher in the samples with high PAFAH1B3 than in those with low PAFAH1B3 (Fig. 3C). In line with these findings, we found that in our 50 clinical HCC samples, patients with high PAFAH1B3 expression had a larger percentage of CD8 + T cells infiltrated than those with low PAFAH1B3 expression (Fig. 3D), and expression of all TEX marker genes was considerably higher in these patients (Fig. 3E), and the expression of PAFAH1B3 was significantly and positively correlated with the expression of TEX-related marker genes (Fig. 3F). Ultimately, an immunofluorescence experiment using double-labeling revealed that in HCC tissues, PAFAH1B3 co-localized mostly with PD1 (Fig. 3G). These all suggest that PAFAH1B3 is closely associated with TEX infiltration in HCC tissues.

### PAFAH1B3 expression was increased in TEX in the single-cell RNA-seq data

**Fig. 4:**
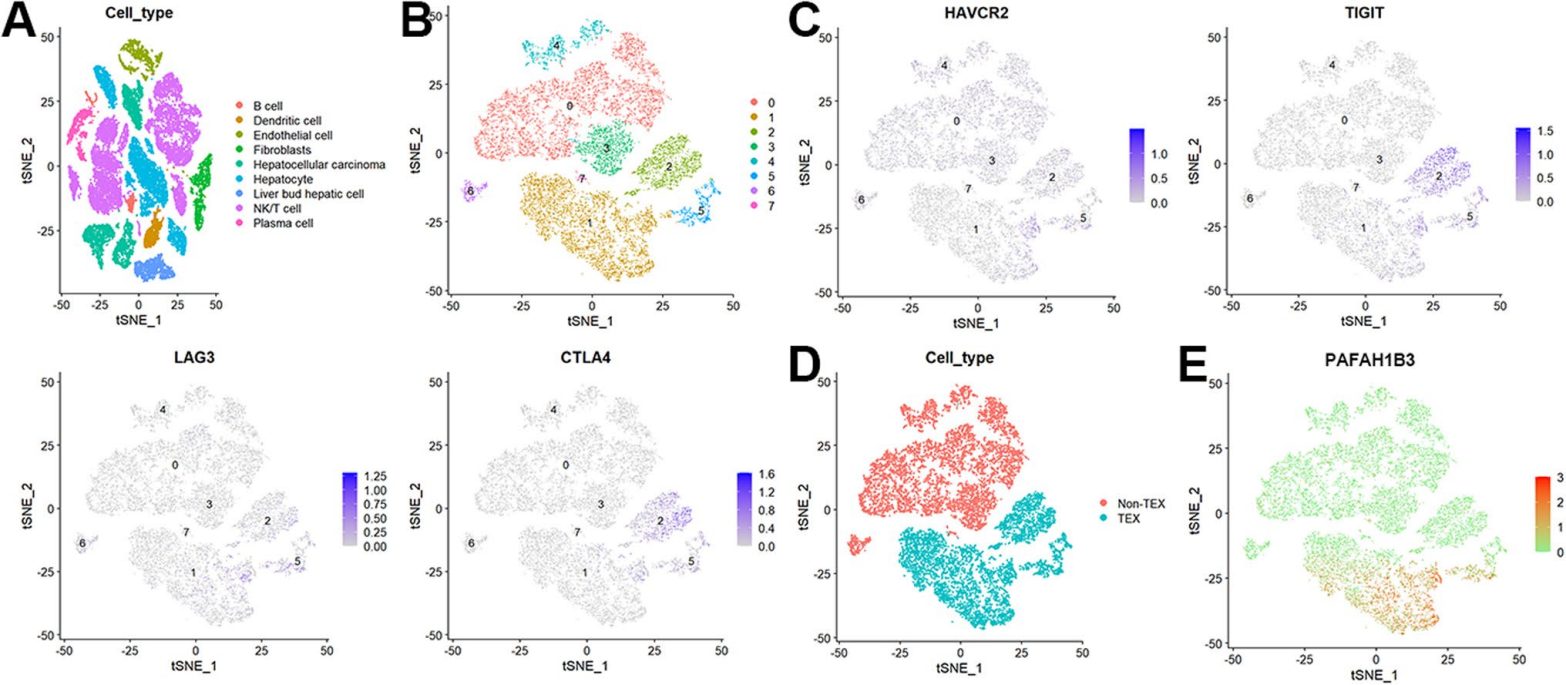
PAFAH1B3 expression was increased in TEX in the single-cell RNA-seq data. **A** Nine cell subpopulations were found in the GSE149614 dataset. **B** Individual NK/T cells were chosen, and they were subpopulated once again. **C** The TEX marker genes. **D** TEX and non-TEX subpopulations. (E)TEX dramatically upregulated PAFAH1B3 expression

Nine cell subpopulations were found in the GSE149614 dataset following the annotation of the 17 distinct subgroups (Fig. 4A). Individual NK/T cells were chosen, and they were subpopulated once again at dim = 50 and resolution = 0.1 (Fig. 4B). After that, based on the expression of the TEX marker genes (Fig. 4C), we distinguished TEX and non-TEX subpopulations in the eight newly discovered subpopulations (Fig. 4D). When compared to non-TEX, TEX dramatically upregulated PAFAH1B3 expression (Fig. 4E).

### Tumor-sensitive drugs targeting PAFAH1B3 prediction

**Fig. 5:**
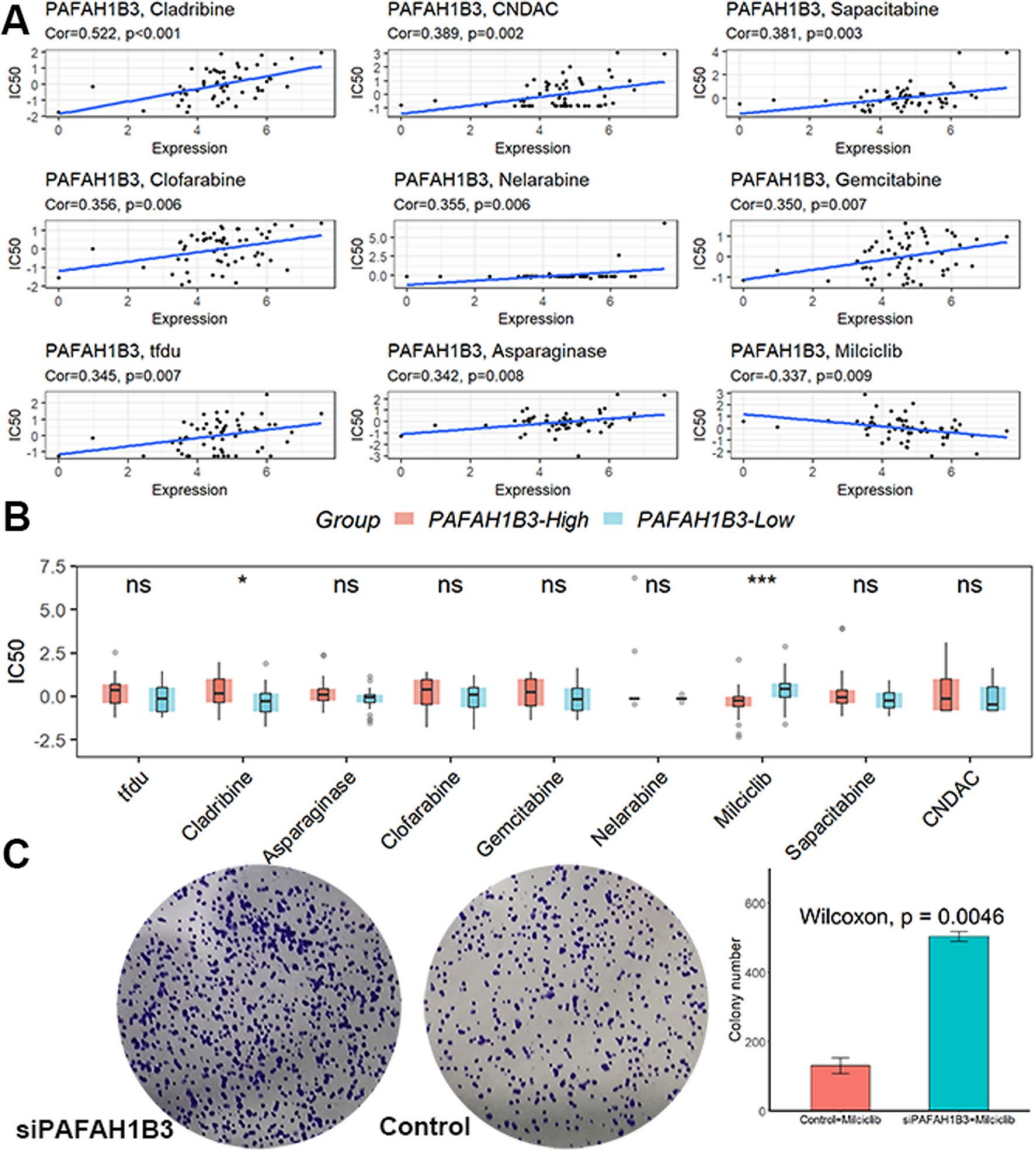
Milciclib may be a tumor-sensitive drug targeting PAFAH1B3. **A** Nine tumor-sensitive drugs targeting PAFAH1B3. **B** Milciclib had lower IC50 values in patients with high PAFAH1B3 expression. **C** Milciclib had a greater effect on the cell proliferation capacity of control cells compared to siPAFAH1B3 cells. ns, not significant. *p < 0.05; ***p < 0.001

Based on the results of the CellMiner analysis, which allowed us to identify nine tumor-sensitive drugs (Fig. 5A), Milciclib had lower IC50 values in patients with high PAFAH1B3 expression (Fig. 5B). In addition, Milciclib had a greater effect on the cell proliferation capacity of control cells compared to siPAFAH1B3 cells, as shown by the results of the colony formation assay (Fig. 5C). All these suggested that Milciclib may be more effective for antitumor therapy in patients with high PAFAH1B3 expression.

### Role of PAFAH1B3 in predicting PD1/PD-L1 immunotherapy

**Fig. 6:**
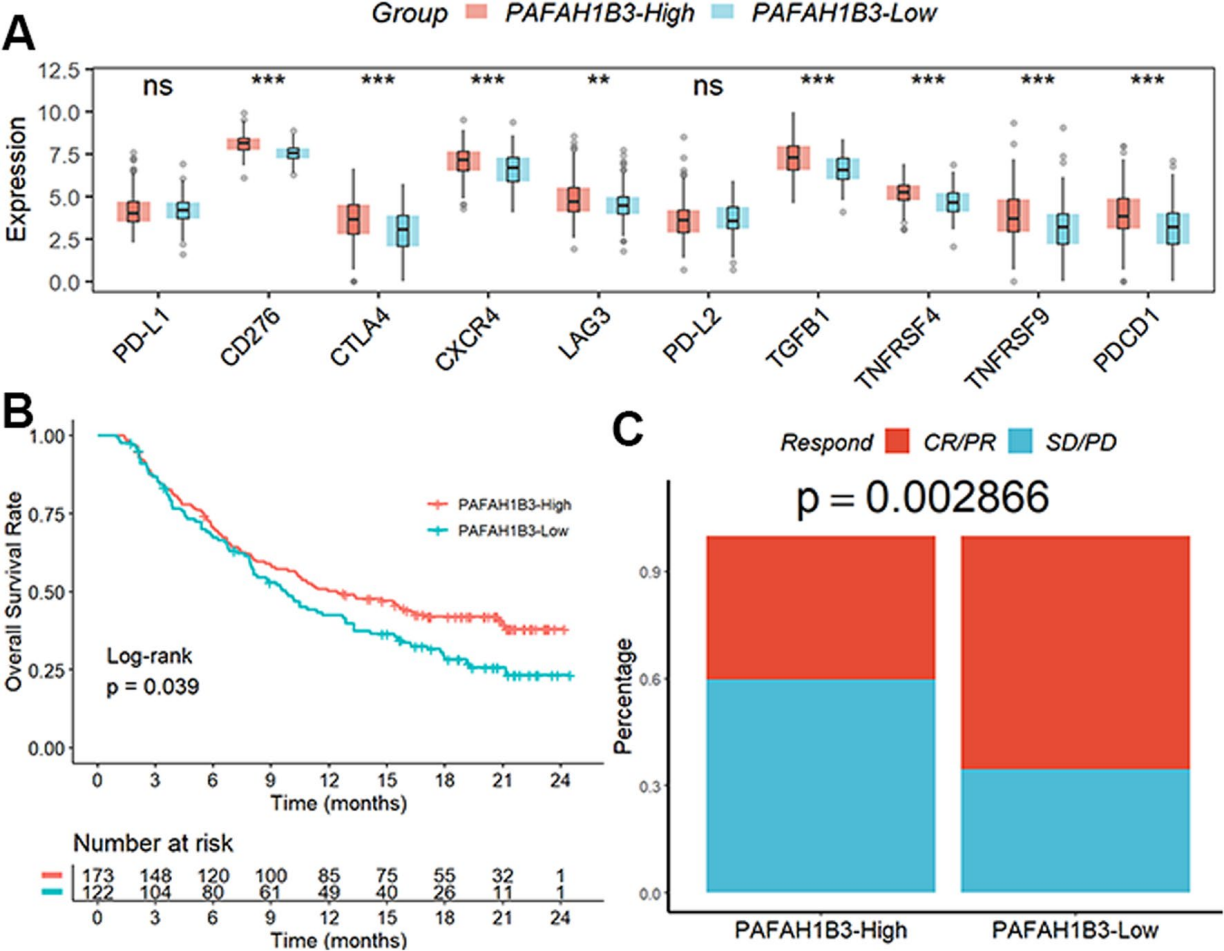
Role of PAFAH1B3 in predicting PD1/PD-L1 immunotherapy. **A** Except for PD-L1 and PD-L2, the majority of ICIs genes were upregulated in patients with elevated PAFAH1B3 expression. **B**, **C** Patients with high PAFAH1B3 expression had a better prognosis and were more likely to respond favorably to PD1/PD-L1 treatment. ns, not significant. **p < 0.01; ***p < 0.001

Figure 6A illustrates our finding that, except for PD-L1 and PD-L2, the majority of ICIs genes were upregulated in patients with elevated PAFAH1B3 expression. We also evaluated PAFAH1B3's predictive capabilities in the IMvigor210 cohort that received PD1/PD-L1 therapy. Patients with high PAFAH1B3 expression had a better prognosis (Fig. 6B) and were more likely to respond favorably to PD1/PD-L1 treatment (Fig. 6C).

## Discussion

PAFAH1B3 has been demonstrated to be involved in regulating the development of a variety of cancers. The expression of PAFAH1B3 was increased in HCC tumor tissue [18], and the effectiveness of treatment is enhanced by PAFAH1B3 blockade, which reduces the proliferation of HCC tumor cells [12, 19]. In this study, we first discovered that PAFAH1B3 acts as a helpful biomarker for PD1/PD-L1 treatment in addition to being strongly related to TEX infiltration.

The critical role played by CD8 + T cells in the control of tumor growth and metastasis is progressively diminished after prolonged exposure to tumor antigens, a process known as TEX [20]. Reduced production of effector cytokines and enhanced expression of inhibitory immunological checkpoint receptors are characteristics that exhausted CD8 + T cells frequently display [21]. Inhibiting TCR expression by attaching to its ligand, PD-L1, the persistently high expression of PD1 on the surface of TEX may reduce immune cell activity. By considerably enhancing immune activity in many cancer patients, blocking PD1/PD-L1 immunosuppression with certain antibodies might revolutionize clinical cancer treatment [22]. In our research, we discovered that patients with greater levels of PAFAH1B3 had a larger percentage of TEX infiltration and considerably higher levels of TEX marker gene expression, indicating a strong intrinsic relationship between PAFAH1B3 and TEX and suggesting that PAFAH1B3 may be a prospective immunotherapy target. Meanwhile, to explore how PAFAH1B3 affects TEX, we first found that the differential genes between two subgroups, PAFAH1B3^High^ and PAFAH1B3^Low^, were mainly enriched in the HALLMARK TNFA SIGNALING VIA NFKB signaling pathway by GSEA analysis (Figure S4), suggesting that PAFAH1B3 may promote the proportion of TEX infiltration through the TNF -α/NF-κB signaling pathway to promote an increased proportion of TEX infiltration. In colon cancer, phosphorylation of NF-κB can promote CD8 + T-cell exhaustion by activating the expression of PD-L1 on the surface of tumor cells [23]. These suggest that PAFAH1B3 may affect TEX through the TNF-α/NF-κB signaling pathway. Of course, this needs to be validated by a large number of ex vivo and in vivo experiments in future work.

Phase II clinical trials are currently being conducted for Milciclib, a small molecule anticancer drug that prevents the formation of complexes between cell cycle protein-dependent kinase 2 and cell cycle protein A and pro-myosin receptor kinase A [24]. Additionally, Milciclib has antagonistic effects on glucose metabolism and microangiogenesis in tumor cells [25, 26]. Milciclib can be a better treatment for patients with thymoma and thymic cancer and has been approved by the U.S. Food and Drug Administration (FDA), and is undergoing phase II clinical studies in HCC to determine its efficacy as a single agent in the treatment of HCC [24]. In this study, we found that Milciclib had lower IC50 values in patients with high PAFAH1B3 expression and had a greater effect on the cell proliferation capacity of control cells compared to siPAFAH1B3 cells, suggesting that Milciclib may be more effective for antitumor therapy in patients with high PAFAH1B3 expression.

Immune checkpoint blockade (ICB) therapies that target PD1 and its ligand PD-L1 have completely changed how cancer is treated and can considerably increase survival rates in a variety of tumor types [27]. Importantly, ICB treatment enhanced the survival outcomes of HCC patients with elevated PAFAH1B3 compared to those with decreased PAFAH1B3. These results emphasize the significance of PAFAH1B3 as a possible immunotherapy biomarker in HCC patients. Although PD-L1 expression correlates with the efficacy of PD-1/PD-L1 inhibitors, the use of PD-L1 as a marker to analyze therapeutic efficacy is ultimately required to obtain validation of clinical drug efficacy. Although PD-L1 expression levels did not vary significantly between patients with high or low PAFAH1B3 expression, in a subsequent cohort of the IMvigor210 cohort, we found that patients with high PAFAH1B3 expression had a significantly better survival outcome after anti-PD-L1 therapy than patients with low PAFAH1B3 expression. Of course, the ability of PAFAH1B3 to serve as a predictive marker for the efficacy of PD-1/PD-L1 inhibitors in HCC patients still needs to be conclusively confirmed in the cohort of HCC patients we recruited to receive PD-L1 or PD1 therapy. In further research, we will conduct more in vivo and in vitro investigations to better understand the mechanism by which PAFAH1B3 influences TEX infiltration to enhance the development of HCC.

## Conclusion

In summary, we found for the first time that PAFAH1B3 is closely associated with TEX and may serve as an effective ICB treatment indicator in HCC.

### Supplementary Information

Additional file1 (DOCX 1134 KB)

Additional file2 (XLSX 15 KB)

Additional file3 (XLSX 20 KB)

## Data Availability

The datasets used and/or analyzed during the current study (TCGA-LIHC, ICGC-LIRI-JP, GSE149614, and IMvigor210 cohorts) are available from the corresponding author upon reasonable request.

## Abbreviations

TEX: T-cell exhaustion
HCC: Hepatocellular carcinoma
IHC: Immunohistochemistry
ImmuCellAI: Immunocell abundance identifier
TME: Tumor microenvironment
PAFAH1B3: Platelet-activating factor acetylhydrolase 1B3
TCGA: The Cancer Genome Atlas
ICIs: Immune checkpoint inhibitors
FDR: False discovery rate
AFP: Alpha-fetoprotein
ICB: Immune checkpoint blockade
FDA: Food and Drug Administration
