# Supplementary material for: Increased PAFAH1B3 was associated with poor prognosis and T-cell exhaustion microenvironment in hepatocellular carcinoma
Source: Discov Oncol. 2023 Dec 8;14:227. doi: 10.1007/s12672-023-00845-6 (PMC10709286; doi:10.1007/s12672-023-00845-6)
Supplement: Supplementary file 1 — Additional file1 (DOCX 1134 KB) [file 12672_2023_845_MOESM1_ESM.docx]

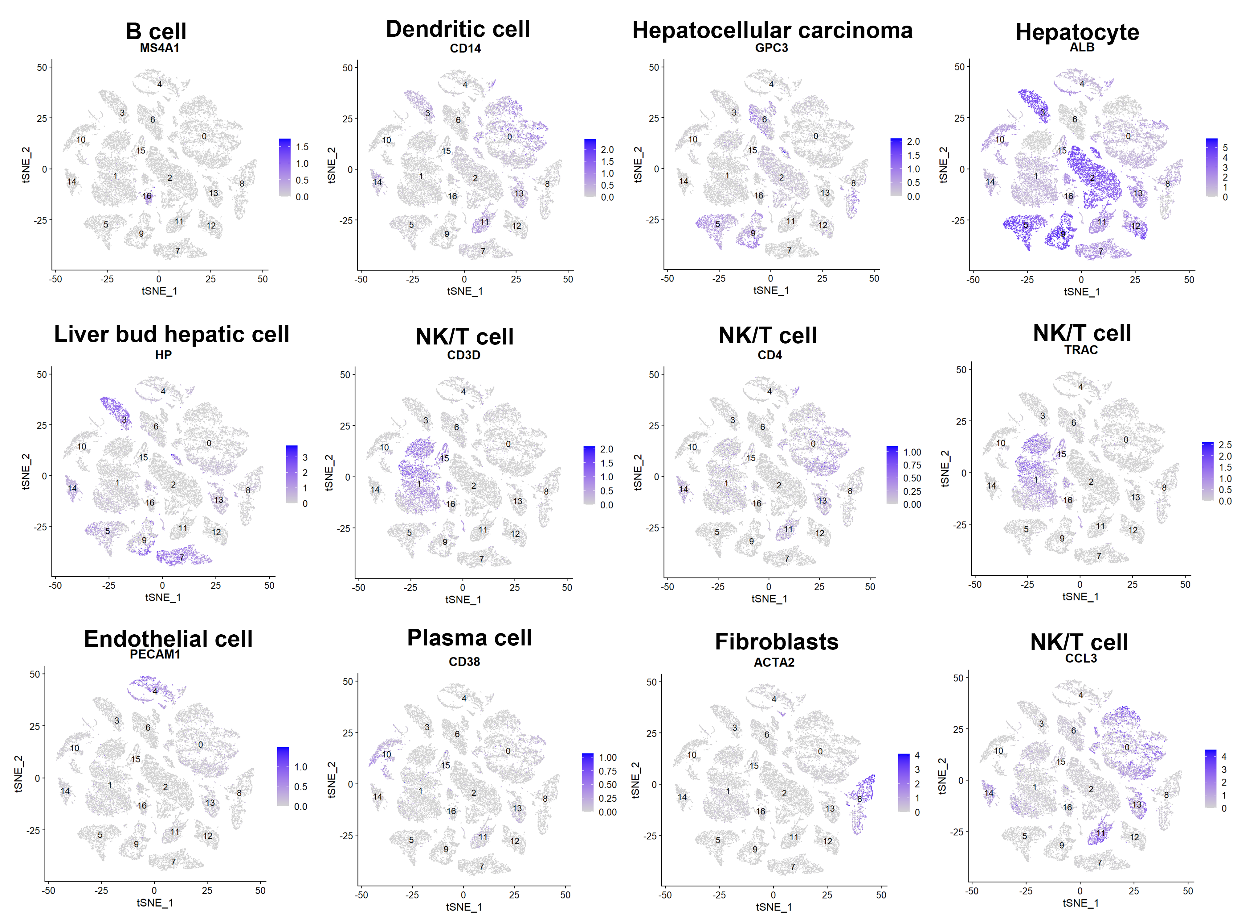


Figure S1 These 17 independent subgroups were annotated according to different marker genes.


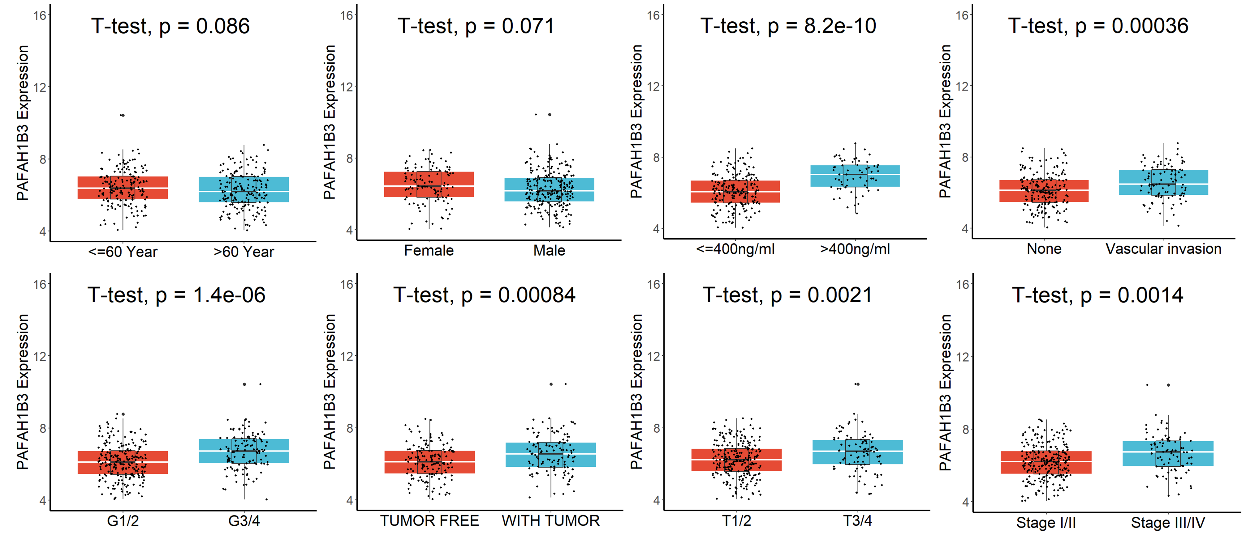


Figure S2 High alpha-fetoprotein (AFP) levels, vascular invasion, later grade, later TNM stage, and recurrence were all substantially correlated with elevated PAFAH1B3 in HCC patients.


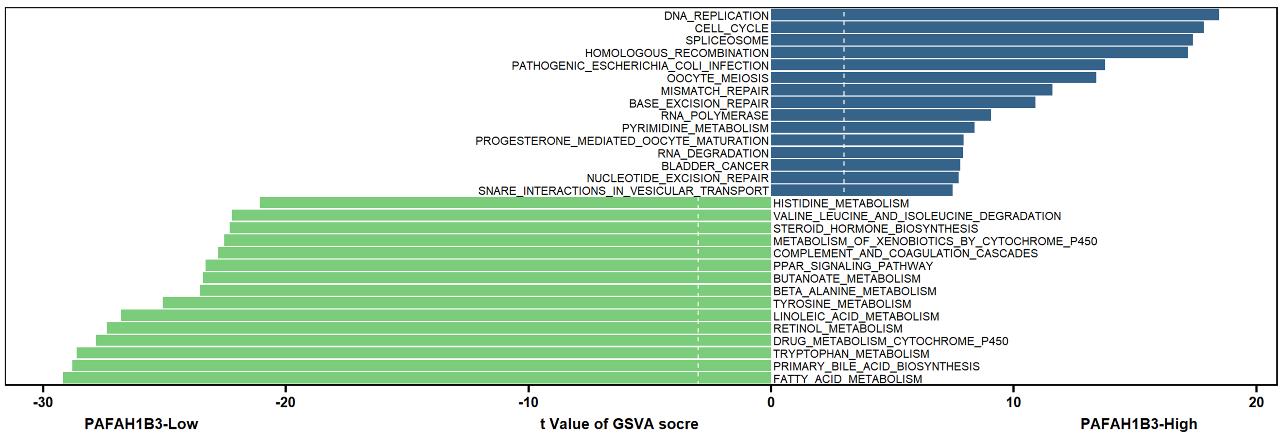


Figure S3 GSVA analysis.


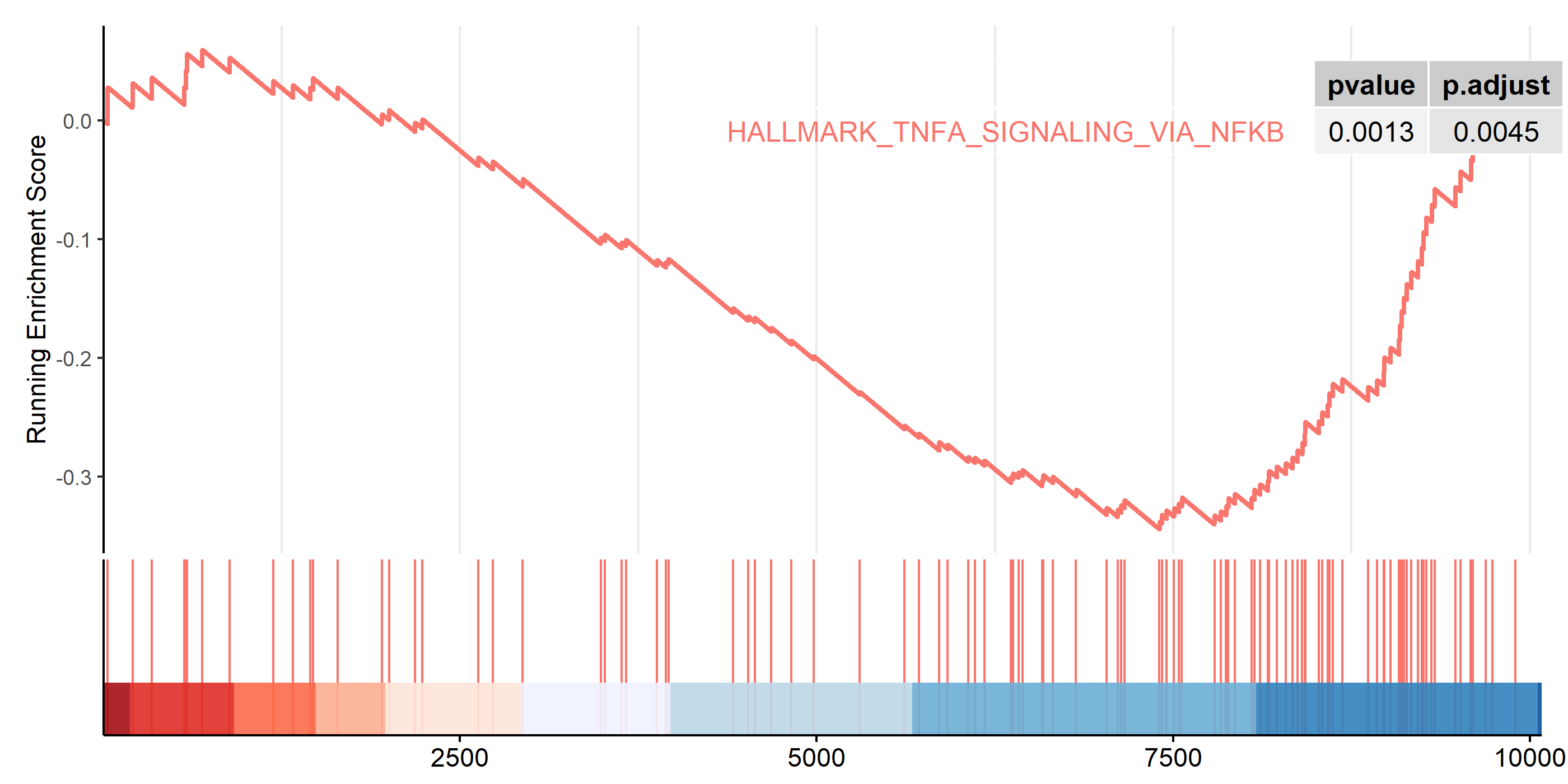


Figure S4 GSEA analysis.

Table S1 Clinical characteristics of HCC patients involved in the study

|  | TCGA cohort  (N=342) | ICGC cohort  (N=227) |
| --- | --- | --- |
| Gender Male | 233 | 61 |
| Female | 109 | 166 |
| Age ≤60 years | 165 | 49 |
| >60 years | 177 | 178 |
| Grade G1/2 | 214 |  |
| G3/4 | 123 |  |
| unknown | 5 |  |
| TNM Stage I/II | 238 | 140 |
| III/IV | 83 | 87 |
| unknown | 21 | 0 |
| Vascular Invasion Yes | 101 |  |
| No | 187 |  |
| unknown | 54 |  |
| Recurrence With tumor | 122 |  |
| Tumor free | 153 |  |
| unknown | 67 |  |
| Cirrhosis With | 65 |  |
| Without | 134 |  |
| unknown | 143 |  |
| AFP value <=400 ng/ml | 199 |  |
| > 400ng/ml | 61 |  |
| NA | 82 |  |

Table S2 The sequences of the qRT-PCR primers used in this study

| Gene | Forward primer | Reverse primer |
| --- | --- | --- |
| PAFAH1B3 | ACATCCGGCCCAAGATTGTG | GGGCTGTCGCTCATTCACC |
| HAVCR2 | CTGCTGCTACTACTTACAAGGTC | GCAGGGCAGATAGGCATTCT |
| TIGIT | TCTGCATCTATCACACCTACCC | CCACCACGATGACTGCTGT |
| CTLA4 | GCCCTGCACTCTCCTGTTTTT | GGTTGCCGCACAGACTTCA |
| LAG3 | GCGGGGACTTCTCGCTATG | GGCTCTGAGAGATCCTGGGG |
| PDCD1 | CCAGGATGGTTCTTAGACTCCC | TTTAGCACGAAGCTCTCCGAT |
| β-ACTIN | CGTGGGCCGCCCTAGGCACCA | TTGGCTTAGGGTTCAGGGGGG |
